# Supplementary material for: Prime editing with genuine Cas9 nickases minimizes unwanted indels
Source: Nat Commun. 2023 Mar 30;14:1786. doi: 10.1038/s41467-023-37507-8 (PMC10063541; doi:10.1038/s41467-023-37507-8)
Supplement: Supplementary file 1 — Supplementary Information [file 41467_2023_37507_MOESM1_ESM.pdf]

Supplementary Information

Prime editing with genuine Cas9 nickases minimizes unwanted indels

Table of Contents

Supplementary Figures

Supplementary Figure 1. nCas9 (H840A) can create DSBs at target sites in the genome
Supplementary Figure 2. Editing frequency of nCas9 variants and PE2 variants at off-target sites
Supplementary Figure 3. Additional mutations in the nCas9 HNH domain for reduction of DSBs induced by nCas9 (H840A)
Supplementary Figure 4. Effect of incorporating HNH domain-deleted nCas9 variants into PE2
Supplementary Figure 5. Deletion frequencies between two gRNA (pegRNA and nicking sgRNA) targeted sites
Supplementary Figure 6. PE variants that incorporate improved nCas9 variants, used in the PE3 system with epegRNAs, achieve higher frequencies of substitutions
Supplementary Figure 7. Average frequencies of correct edits and unwanted indels induced by PE variants that incorporate improved nCas9 variants, used in the PE3 system with epegRNAs encoding substitutions, insertions, and deletions
Supplementary Figure 8. epegRNAs used together with PE variants that incorporate nCas9 (H840A+N863A) or nCas9 (H840A+N854A) increase the purity of the correct edit for the PE3 system in K562 cells
Supplementary Figure 9. epegRNAs used together with PE variants that incorporate nCas9 (H840A+N863A) or nCas9 (H840A+N854A) increase the purity of the correct edit for the PE3 system in HeLa cells

Supplementary Tables

(Please find attached Supplementary Tables file.)

Supplementary Table 1. List of primers used for targeted deep sequencing

Supplementary Table 2. Sequences of pegRNAs for PE2 experiments

Supplementary Table 3. Sequences of pegRNAs and sgRNAs for PE3 experiments

Supplementary Table 4. Sequences of epegRNAs and sgRNAs for single base substitution, Flag-tag insertion and 15-bp deletion used in mammalian cell experiments

Supplementary Table 5. DNA sequences of plasmids used in experiments

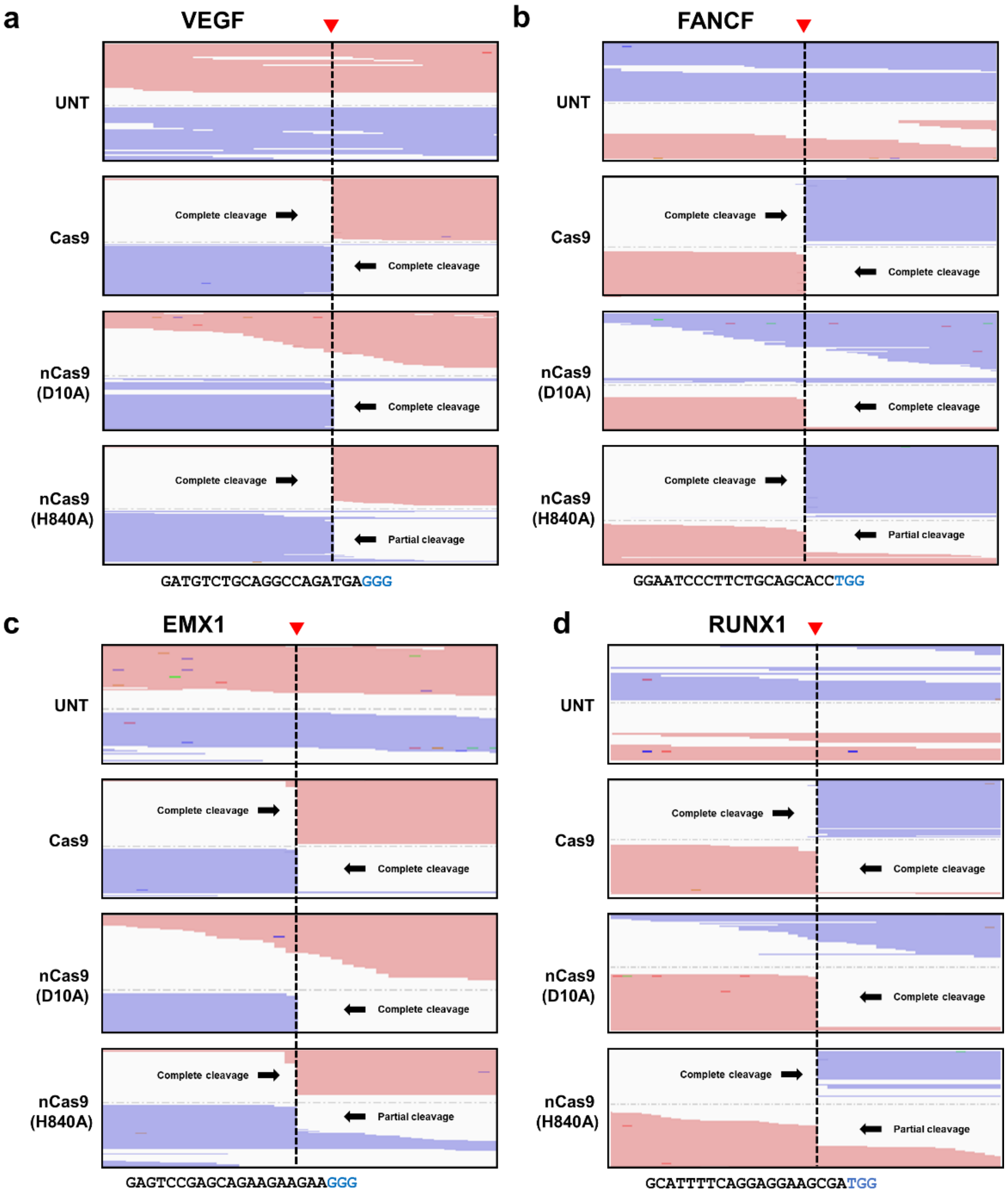

Supplementary Figure 1. nCas9 (H840A) can create DSBs at target sites in the genome a-d, Genomic DNAs isolated from HEK293T cells were treated in vitro with purified WT Cas9, nCas9 (D10A), or nCas9 (H840A) proteins, together with in vitro transcribed sgRNAs targeted to four different sites, and then subjected to WGS. The resulting cleavage patterns at the *VEGFA* (a), *FANCF* (b), *EMX1* (c), and *RUNX1* (d) sites visualized using the IGV viewer. Red arrow head: cleavage site. Blue characters: PAM sequence.

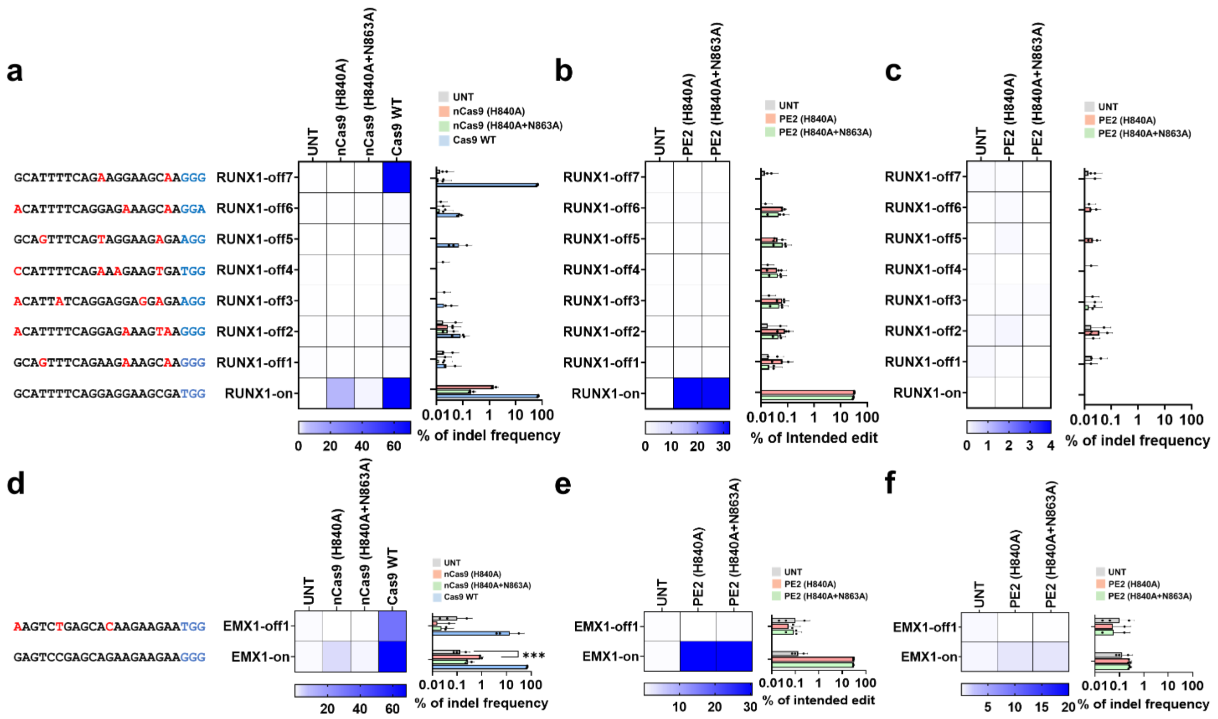

Supplementary Figure 2. Editing frequency of nCas9 variants and PE2 variants at off-target sites

Genomic DNAs of HEK293T cells treated with nCas9 (H840A), nCas9 (H840A+N863A), WT Cas9, PE2 (H840A) and PE (H840A+N863A) were isolated and measured the editing frequencies at selected off-target sites. Off-target sites were captured by Digenome-seq (nCas9 (H840A)-induced DSB sites) and selected under conditions of the digenome score (≥ 8.0) and the number of mismatched bases (≤ 6bp). **a**, Indel frequencies of nCas9 (H840A), nCas9 (H840A+N863A), and WT Cas9 at the *RUNX1* on- and off-target sites were measured by targeted deep sequencing. **b,c,** Intended edits (b) and unwanted indel frequencies (c) of PE2 (H840A) and PE (H840A+N863A) at *RUNX1* on- and off-target sites were measured. **d,** Indel frequencies of nCas9 (H840A), nCas9 (H840A+N863A), and WT Cas9 at *EMX1* on- and off-target sites were calculated. ***; p=0.000498. **e,f,** Intended edits (e) and unwanted indel frequencies (f) of PE2 (H840A) and PE (H840A+N863A) at *EMX1* on- and off-target sites were calculated. Mismatched sequence (red) and PAM sequence (blue). Heat map colors and means ± SEM (**a-f**) were determined from three independent experiments. All statistical analysis for samples were conducted using unpaired Student's t-test (two-tailed) in GraphPad Prism 8. (ns, not significant, *p<0.05, **p<0.01, and ***: p<0.001 by student's t test). Source data are provided as a Source Data file.

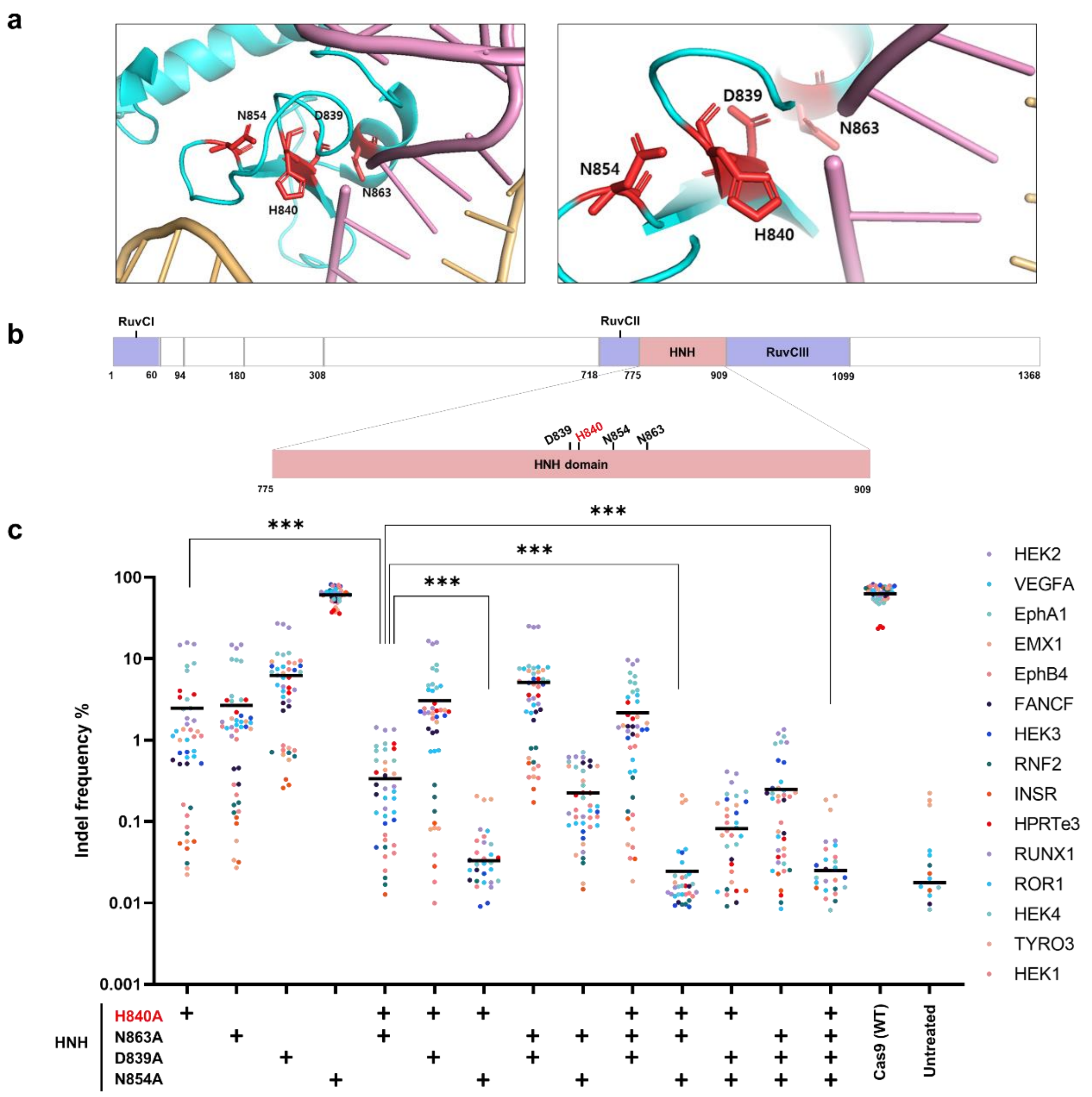

Supplementary Figure 3. Additional mutations in the nCas9 HNH domain for reduction of DSBs induced by nCas9 (H840A)

a, Cryo-EM structure of a portion of SpCas9 in the cleavage state (state II). Candidate amino acids are indicated in red. Pink: DNA; yellow: sgRNA (PBD:6O0Y). **b,** Location of candidate amino acids (D839, N854, and N863) for mutagenesis in the Cas9 sequence. **c,** Plasmids encoding Cas9 and nCas9 variants containing different amino acid mutations and individual sgRNAs targeting 15 endogenous sites were transfected into HEK293T cells and the average indel frequencies at the 15 different loci were measured by targeted deep-sequencing. From left, 1st ***; $p = 0.000606$, 2nd ***; $p = 0.000002$, 3rd ***; $p < 0.000001$ and 4th ***; $p < 0.000001$. Mean (**c**) of all individual values of sets of n =3 independent replicates are shown. All statistical analysis for samples were conducted using unpaired Student's t-test (two-tailed) in GraphPad Prism 8. (ns, not significant, *p<0.05, **p<0.01, and ***: p<0.001 by student's t test). Source data are provided as a Source Data file.

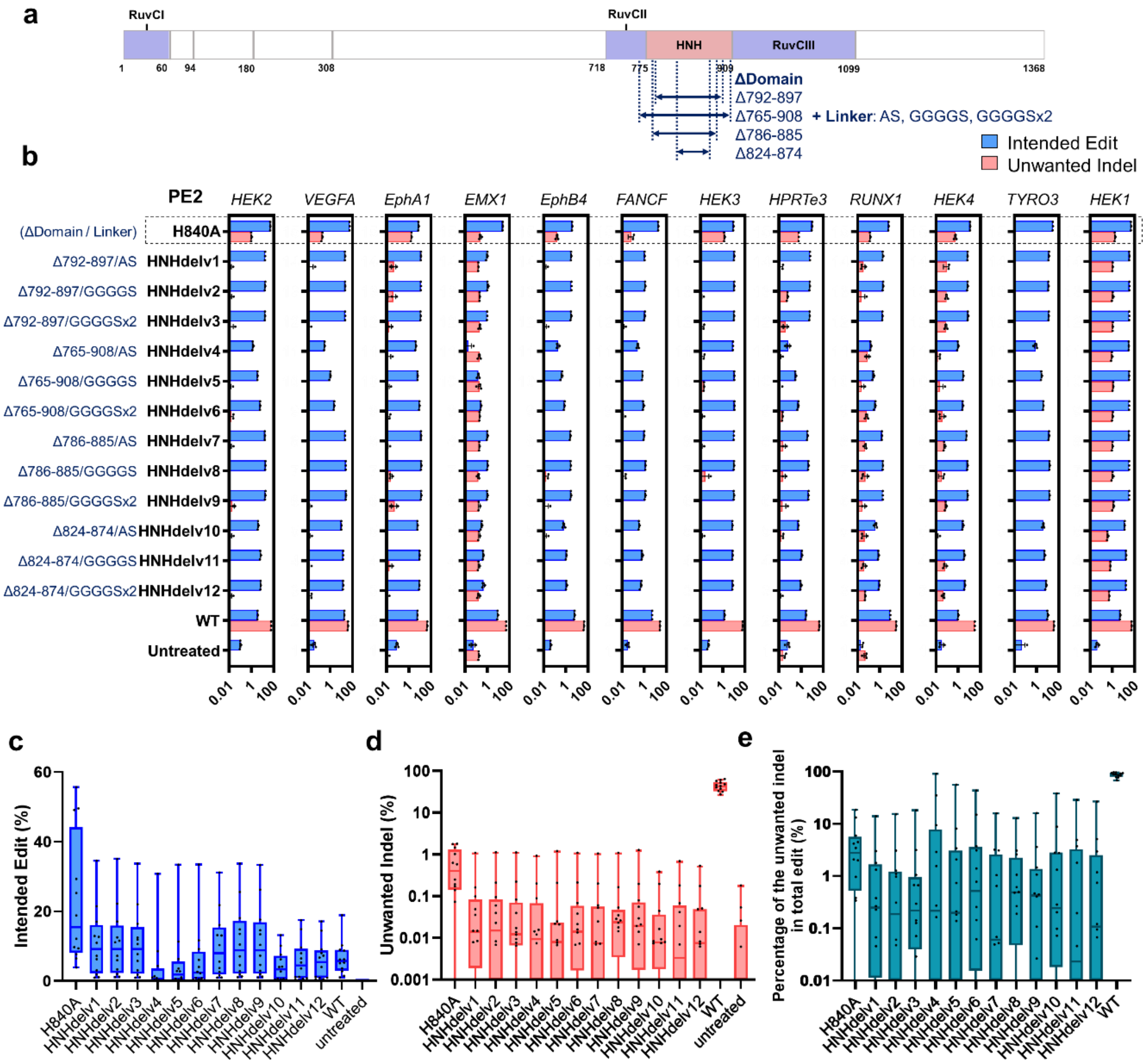

Supplementary Figure 4. Effect of incorporating HNH domain-deleted nCas9 variants into PE2

a, Schematic diagram of HNH-deleted nCas9 variant constructs. **b,** Plasmids encoding PE (HNH-deleted nCas9 variants) and individual pegRNAs targeting 12 endogenous loci were transfected into HEK293T cells. Frequencies of intended edits and unwanted indels were determined for each site. The deletion-linker combinations that were used are indicated to the left of the graphs. **c-d,** Average frequencies of intended edits (c) and unwanted indels (d). **e,** The percentage of unwanted indels among all edited sequences (unwanted indels + intended edits) for the 12 target sites. Mean ± SEM (**b**) were determined three independent experiments. Mean ± SEM (**c, d, e**) of all individual values of sets of n =3 independent replicates were shown. For the boxes (**c, d, e**), the top, middle, and bottom lines represent the 25th, 50th, and 75th percentiles, respectively. The whiskers indicate min to max values. Source data are provided as a Source Data file.

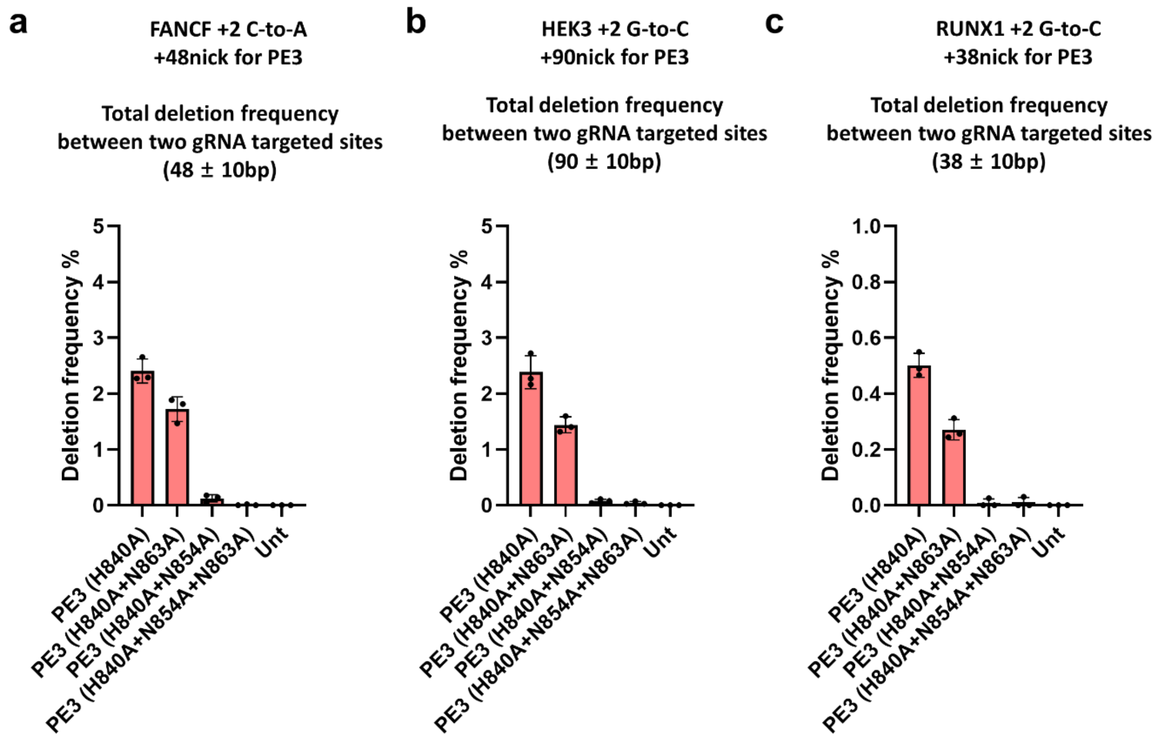

Supplementary Figure 5. Deletion frequencies between two gRNA (pegRNA and nicking sgRNA) targeted sites

Aligned sequences that contained deletions with a length that was ± 10bp of the distance between the two potential DSBs were examined. The distance between pegRNA and nicking sgRNA used for PE3 system in each target site were 48bp, 90bp and 38bp for *FANCF, HEK3* and *RUNX1* site, respectively. **a-c,** Total deletion frequencies of *FANCF* (a), *HEK3* (b) and *RUNX1*(c) targeted samples were calculated. Mean ± SEM (**a,b,c**) were determined from three independent experiment. Source data are provided as a Source Data file.

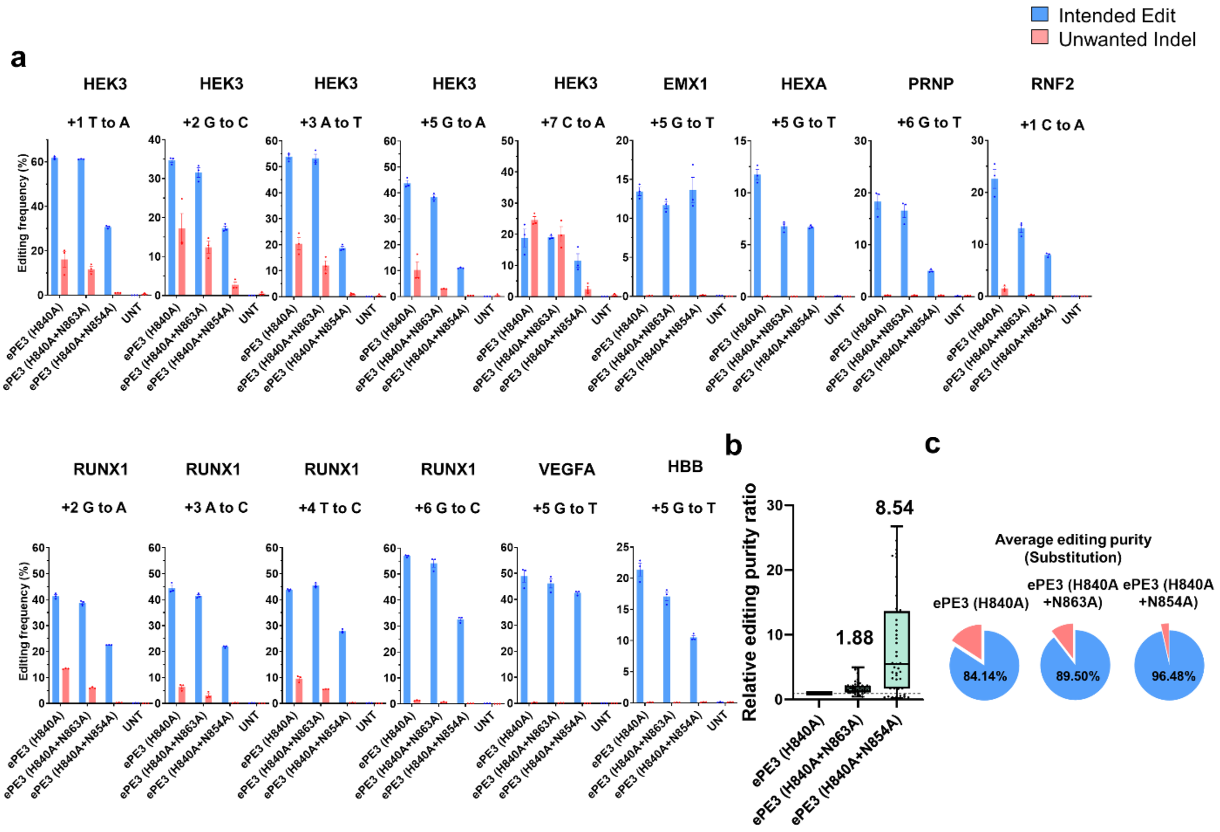

Supplementary Figure 6. PE variants that incorporate improved nCas9 variants, used in the PE3 system with epegRNAs, achieve higher frequencies of substitutions

Plasmids encoding PE (nCas9 variants), individual epegRNAs encoding single-base substitutions targeted to the *HEK3, EMX1, HEXA, PRNP, RNF2, RUNX1, VEGFA* and *HBB* sites, and nicking sgRNAs were transfected into HEK293T cells and their editing efficiencies were examined by targeted-deep sequencing. **a,** The intended base substitutions were indicated on the top of each graphs. **b,** Relative editing purity ratios normalized to the ePE3 (H840A) activity for each site. **c,** The average editing purities for sigle-base substitutions in HEK293T cells shown in pie charts. Blue: intended edits; red: unwanted indels. Average editing purity: the number of reads containing the correct edit / the total number of reads containing edits (correct edits + unwanted edits) *100. Mean ± SEM (**a**) were determined three independent experiments. Mean ± SEM (**b**) of all individual values of sets of n =3 independent replicates were shown. For the boxes (**b**), the top, middle, and bottom lines represent the 25th, 50th, and 75th percentiles, respectively. The whiskers indicate min to max values. Source data are provided as a Source Data file.

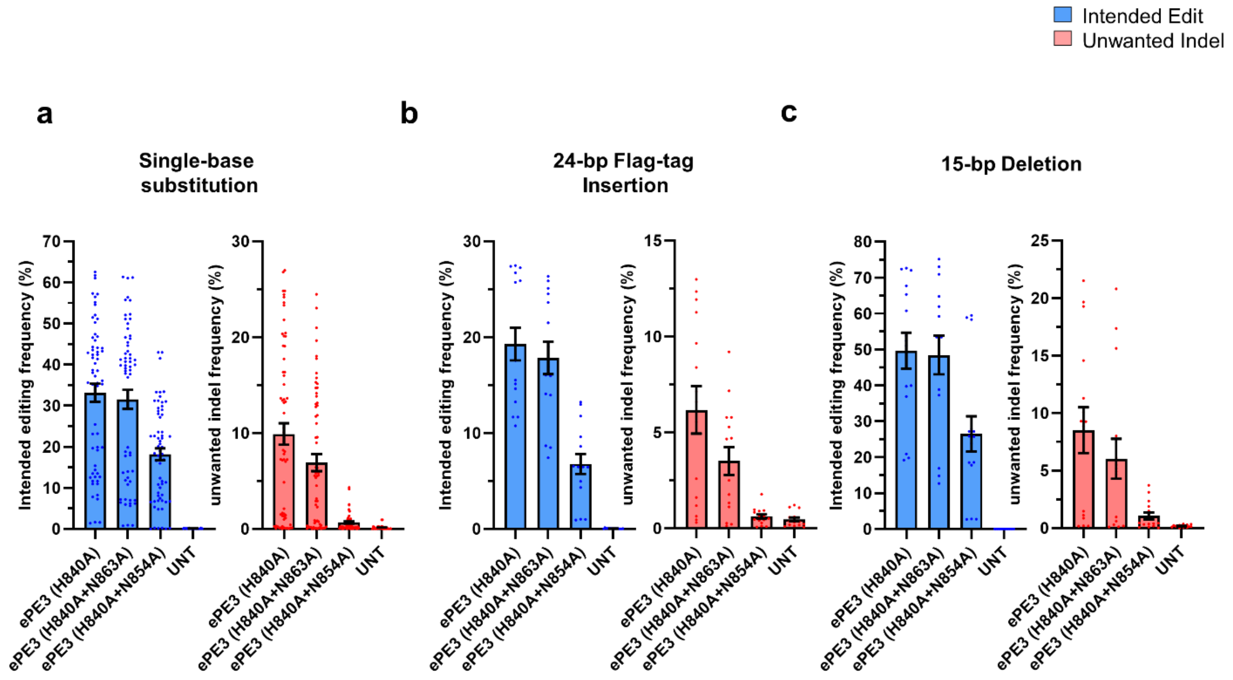

Supplementary Figure 7. Average frequencies of correct edits and unwanted indels induced by PE variants that incorporate improved nCas9 variants, used in the PE3 system with epegRNAs encoding substitutions, insertions, and deletions

a-c, Plasmids encoding PE (nCas9 variants), individual epegRNAs encoding a single-base substitution targeted to the 9 genomic sites (a), or a 24-bp Flag-tag insertion (b) or a 15-bp deletion (c) in the *HEK3, FANCF, VEGFA, RUNX1* and *RNF2* sites, and nicking sgRNAs were transfected into HEK293T cells. Average frequencies of intended edits and unwanted indels are shown. Mean ± SEM (**a, b, c**) of all individual values of sets of n =3 independent replicates were shown. Source data are provided as a Source Data file.

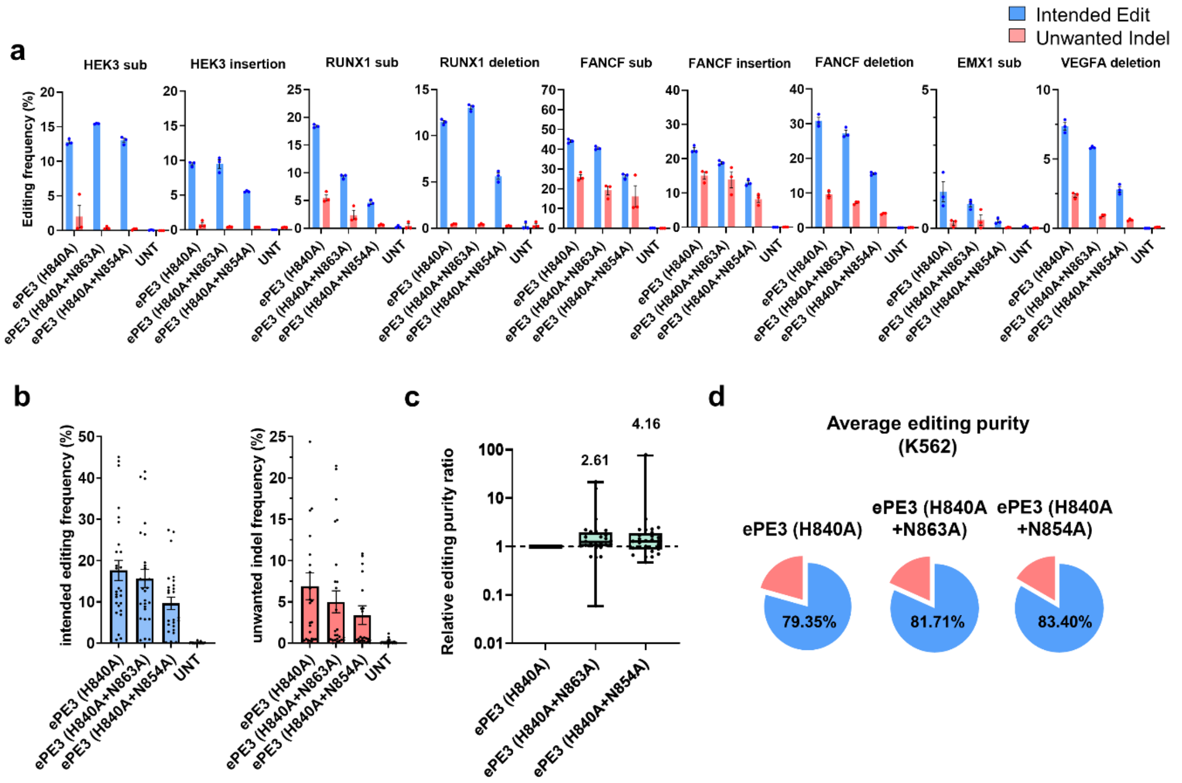

Supplementary Figure 8. epegRNAs used together with PE variants that incorporate nCas9 (H840A+N863A) or nCas9 (H840A+N854A) increase the purity of the correct edit for the PE3 system in K562 cells

Plasmids encoding PE (nCas9 variants), individual epegRNAs encoding single-base substitutions, Flag-tag insertion and 15-bp deletion targeted to the *HEK3, RUNX1, FANCF, EMX1* and *VEGFA* sites, and nicking sgRNAs were transfected into K562 cells. **a-b,** Editing outcomes for each sites (a) and average editing frequencies of intended edit and unwanted indels (b) are shown**. c,** Relative editing purity ratios normalized to the ePE3 (H840A) activity for each site. **d,** The average editing purities of ePE3 variants in K562 cells shown in pie charts. Blue: intended edits; red: unwanted indels. Average editing purity [the number of reads containing the correct edit / the total number of reads containing edits (correct edits + unwanted edits) *100] were calculated. Mean ± SEM (**a**) were determined three independent experiments. Mean ± SEM (**b, c**) of all individual values of sets of n =3 independent replicates were shown. For the boxes (**c**), the top, middle, and bottom lines represent the 25th, 50th, and 75th percentiles, respectively. The whiskers indicate min to max values. Source data are provided as a Source Data file.

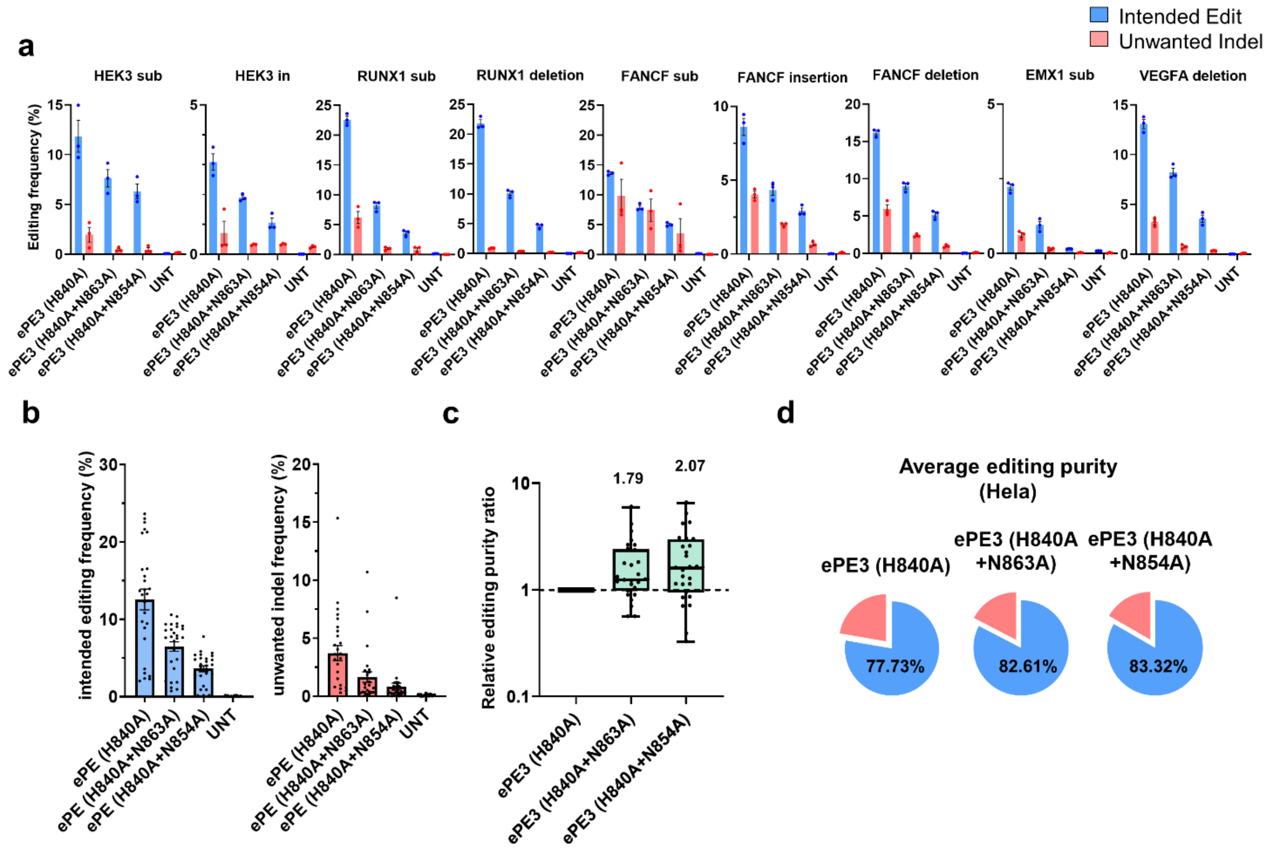

Supplementary Figure 9. epegRNAs used together with PE variants that incorporate nCas9 (H840A+N863A) or nCas9 (H840A+N854A) increase the purity of the correct edit for the PE3 system in HeLa cells.

Plasmids encoding PE (nCas9 variants), individual epegRNAs encoding single-base substitutions, Flag-tag insertion and 15-bp deletion targeted to the *HEK3, RUNX1, FANCF, EMX1* and *VEGFA* sites, and nicking sgRNAs were transfected into HeLa cells. **a-b,** Editing outcomes for each sites (a) and average editing frequencies of intended edit and unwanted indels (b) are shown**. c,** Relative editing purity ratios normalized to the ePE3 (H840A) activity for each site. **d,** The average editing purities of ePE3 variants in HeLa cells shown in pie charts. Blue: intended edits; red: unwanted indels. Average editing purity [the number of reads containing the correct edit / the total number of reads containing edits (correct edits + unwanted edits) *100] were calculated. Mean ± SEM (**a**) were determined three independent experiments. Mean ± SEM (**b, c**) of all individual values of sets of n =3 independent replicates were shown. For the boxes (**c**), the top, middle, and bottom lines represent the 25th, 50th, and 75th percentiles, respectively. The whiskers indicate min to max values. Source data are provided as a Source Data file.
