# Supplementary material for: Prime editing with genuine Cas9 nickases minimizes unwanted indels
Source: Nat Commun. 2023 Mar 30;14:1786. doi: 10.1038/s41467-023-37507-8 (PMC10063541; doi:10.1038/s41467-023-37507-8)
Supplement: Supplementary file 2 — Reporting Summary [file 41467_2023_37507_MOESM2_ESM.pdf]

nature portfolio

Corresponding author(s): Jin-Soo Kim

Last updated by author(s): Mar 13, 2023

Reporting Summary

Nature Portfolio wishes to improve the reproducibility of the work that we publish. This form provides structure for consistency and transparency in reporting. For further information on Nature Portfolio policies, see our Editorial Policies and the Editorial Policy Checklist.

Statistics

For all statistical analyses, confirm that the following items are present in the figure legend, table legend, main text, or Methods section.

n/a	Confirmed	
☐	☒	The exact sample size (n) for each experimental group/condition, given as a discrete number and unit of measurement
☐	☒	A statement on whether measurements were taken from distinct samples or whether the same sample was measured repeatedly
☐	☒	The statistical test(s) used AND whether they are one- or two-sided<br>*Only common tests should be described solely by name; describe more complex techniques in the Methods section.*
☒	☐	A description of all covariates tested
☒	☐	A description of any assumptions or corrections, such as tests of normality and adjustment for multiple comparisons
☐	☒	A full description of the statistical parameters including central tendency (e.g. means) or other basic estimates (e.g. regression coefficient) AND variation (e.g. standard deviation) or associated estimates of uncertainty (e.g. confidence intervals)
☐	☒	For null hypothesis testing, the test statistic (e.g. F, t, r) with confidence intervals, effect sizes, degrees of freedom and P value noted<br>*Give P values as exact values whenever suitable.*
☒	☐	For Bayesian analysis, information on the choice of priors and Markov chain Monte Carlo settings
☒	☐	For hierarchical and complex designs, identification of the appropriate level for tests and full reporting of outcomes
☒	☐	Estimates of effect sizes (e.g. Cohen's d, Pearson's r), indicating how they were calculated

Our web collection on statistics for biologists contains articles on many of the points above.

Software and code

Policy information about availability of computer code

Data collection	The MiniSeq system (Illumina) was used to perform targeted deep sequencing.<br>For structural assay, we used PyMol (2.5.2) and custom Python (3.9) script described in Code availability.
Data analysis	Source code (https://github.com/ibs-cge2/prime_editor_analysis) and (https://github.com/chizksh/digenome-toolkit2) were used to determine prime editing frequency and indel frequency from targeted deep sequencing data and digenome_seq respectively. For data analysis and visualization, we used IGV (2.5.3), Microsoft Excel (2016), Powerpoint (2016), BWA (v.0.7.17), SAMtools (v.1.9), ImageJ (1.53t) and Prism 8 (8.4.3) was used for drawing figures, graphs, and tables.

For manuscripts utilizing custom algorithms or software that are central to the research but not yet described in published literature, software must be made available to editors and reviewers. We strongly encourage code deposition in a community repository (e.g. GitHub). See the Nature Portfolio guidelines for submitting code & software for further information.

Data

Policy information about availability of data

All manuscripts must include a data availability statement. This statement should provide the following information, where applicable:

- Accession codes, unique identifiers, or web links for publicly available datasets
- A description of any restrictions on data availability
- For clinical datasets or third party data, please ensure that the statement adheres to our policy

DNA sequencing data have been deposited in the National Center for Biotechnology Information (NCBI) Sequence Read Archive (SRA) database with BioProject accession code PRJNA888859. The protein-structure data for HNH domain of Cas9 is from Protein Data Bank (https://www.rcsb.org). PDB code is 6O0Y for Cas9 state II (http://doi.org/10.2210/pdb6O0Y/pdb). Source data are provided with this paper.

Human research participants

Policy information about studies involving human research participants and Sex and Gender in Research.

Reporting on sex and gender	N/A
Population characteristics	N/A
Recruitment	N/A
Ethics oversight	N/A

Note that full information on the approval of the study protocol must also be provided in the manuscript.

Field-specific reporting

Please select the one below that is the best fit for your research. If you are not sure, read the appropriate sections before making your selection.

- [x] Life sciences
- [ ] Behavioural & social sciences
- [ ] Ecological, evolutionary & environmental sciences

For a reference copy of the document with all sections, see nature.com/documents/nr-reporting-summary-flat.pdf

Life sciences study design

All studies must disclose on these points even when the disclosure is negative.

Sample size	Sample sizes were determined based on previous publications of our and other groups generating reproducible results for genome editing experiments.
Data exclusions	No data was excluded from the analyses.
Replication	All attempts at replication (n=2 or 3) were successful for cell line experiments.
Randomization	Mammalian cells utilized in the study were grown under identical condition. Thus, samples were not randomized.
Blinding	Mammalian cells utilized in the study were grown under identical condition. Thus, Investigators were not blinded during experiments and data analysis.

Reporting for specific materials, systems and methods

We require information from authors about some types of materials, experimental systems and methods used in many studies. Here, indicate whether each material, system or method listed is relevant to your study. If you are not sure if a list item applies to your research, read the appropriate section before selecting a response.

Materials & experimental systems

n/a	Involved in the study
x	Antibodies
	x Eukaryotic cell lines
x	Palaeontology and archaeology
x	Animals and other organisms
x	Clinical data
x	Dual use research of concern

Methods

n/a	Involved in the study
x	ChIP-seq
x	Flow cytometry
x	MRI-based neuroimaging

Eukaryotic cell lines

Policy information about cell lines and Sex and Gender in Research

Cell line source(s)	HEK293T (CRL-11268, American Type Culture Collection), Hela (CCL-2, American Type Culture Collection) and K562 CCL-243, American Type Culture Collection)
Authentication	No cell lines were authenticated.
Mycoplasma contamination	Cells were not tested for mycoplasma contamination
Commonly misidentified lines (See ICLAC register)	No commonly misidentified lines were used
