# Supplementary material for: Prime editing with genuine Cas9 nickases minimizes unwanted indels
Source: Nat Commun. 2023 Mar 30;14:1786. doi: 10.1038/s41467-023-37507-8 (PMC10063541; doi:10.1038/s41467-023-37507-8)
Supplement: Supplementary file 9 — Description of Additional Supplementary Files [file 41467_2023_37507_MOESM9_ESM.pdf]

Title: Supplementary Data 1.
Description: DNA sequences of plasmids used in experiments
Sequences of plasmids used in this study are provided.

Title: Supplementary Data 2.
Description: Sequences of pegRNAs for PE2 experiments.
Information of pegRNAs used for PE2 experiments are provided.

Title: Supplementary Data 3.
Description: Sequences of pegRNAs and sgRNAs for PE3 experiments.
Information of pegRNAs and nicking sgRNAs used for PE3 experiments are provided.

Title: Supplementary Data 4.
Description: Sequences of epegRNAs and sgRNAs for single base substitution, Flag-tag insertion and 15-bp deletion used in mammalian cell experiments.
Information of epegRNAs and nicking sgRNA used for ePE3 experiments are provided

Title: Supplementary Data 5.
Description: List of primers used for targeted deep sequencing
Primers used for targeted deep sequencing. Spacer sequences, 1st and 2nd PCR primer sequences are provided**.**
